# An Optimized Method for Evaluating the Potential Gd-Nanoparticle Dose Enhancement Produced by Electronic Brachytherapy

**DOI:** 10.3390/nano14050430

**Published:** 2024-02-27

**Authors:** Melani Fuentealba, Alejandro Ferreira, Apolo Salgado, Christopher Vergara, Sergio Díez, Mauricio Santibáñez

**Affiliations:** 1 Departamento de Cs. Físicas, Universidad de La Frontera, Temuco 4811230, Chile; 2 Laboratorio de Radiaciones Ionizantes, Universidad de La Frontera, Temuco 4811230, Chile; 3 Departamento de Fisiología, Universitat de Valencia, 46010 Valencia, Spain; 4 Facultad de Medicina y Ciencia, Universidad San Sebastián, Santiago 7510602, Chile; 5 IORT, Santiago 7500833, Chile; 6 Medical Physics Department, Hospital Clínico Universitario de Valencia, 46010 Valencia, Spain

**Keywords:** dose enhancement, delaminated EBT3 films, Gd nanoparticles

## Abstract

This work reports an optimized method to experimentally quantify the Gd-nanoparticle dose enhancement generated by electronic brachytherapy. The dose enhancement was evaluated considering energy beams of 50 kVp and 70 kVp, determining the Gd-nanoparticle concentration ranges that would optimize the process for each energy. The evaluation was performed using delaminated radiochromic films and a Poly(methyl methacrylate) (PMMA) phantom covered on one side by a thin 2.5 μm Mylar filter acting as an interface between the region with Gd suspension and the radiosensitive film substrate. The results for the 70 kVp beam quality showed dose increments of 6±6%, 22±7%, and 9±7% at different concentrations of 10, 20, and 30 mg/mL, respectively, verifying the competitive mechanisms of enhancement and attenuation. For the 50 kVp beam quality, no increase in dose was recorded for the concentrations studied, indicating that the major contribution to enhancement is from the K-edge interaction. In order to separate the contributions of attenuation and enhancement to the total dose, measurements were replicated with a 12 μm Mylar filter, obtaining a dose enhancement attributable to the K-edge of 29±7% and 34±7% at 20 and 30 mg/mL, respectively, evidencing a significant additional dose proportional to the Gd concentration.

## 1. Introduction

It is widely known that high-atomic-number nanoparticle infusion within biological tissue presents multiple benefits in diagnostic and therapeutic terms, as is the case for teletherapy and brachytherapy [1,2,3,4]. This is due to the localized deposition of energy by photoelectrons and Auger electrons produced by photoelectric interaction augmentation between primary radiation and the high-Z nanoparticle infused into the tissue. This effect is denominated as dose enhancement and has the characteristics of being highly localized and restricted exclusively to the infusion area, increasing the dose solely in the region of interest without affecting the surrounding tissue [5,6,7].

There are several radiotherapy modalities that can potentially produce a relevant dose enhancement, such as: electronic brachytherapy (eBx) [8,9,10], intraoperative radiotherapy (IORT) [11,12,13], and superficial radiation therapy (SRT) [14,15] by low-energy X-rays. These treatment modalities, using new equipment, promise to replace the traditional sources of conventional brachytherapy treatments based on radioactive isotopes (typically ^192^Ir, ^60^Co, and the obsolete ^137^Cs) by the new generations of mini-X-ray tubes with energies in the range of 35–100 kVp [16,17].

The production of nanoparticle agents with high internalization, biocompatibility, and the ability to be incorporated inside malignant cells at a higher concentration compared to normal tissue cells has been studied in detail for metallic nanoparticles such as gold (GNPs). Functionalization through bonding to lipids [18,19], peptides [20,21,22], proteins [23,24], and antibodies associated with over-expression in tumoral cells [6,25,26,27,28] is the path explored to achieve high tumor concentrations. Even though GNPs have been researched the most, gadolinium nanoparticles (GdNPs) are receiving more attention (given their long-standing history in clinical imagenology as safe MRI contrast agents), with experimental studies demonstrating great biocompatibility and internalization capabilities for in vitro and in vivo applications [29,30]. The first investigations that explored the viability of Gd as a dose enhancer assessed its use in treating brain tumors via the application of microbeams [31,32]. GdNPs such as AGuIX^®^ NPs (NH TherAguix SA, Meylan, France) have been used for human studies in MRI-guided radiotherapy [29], as well as phase-I clinical trials [33,34] as dose enhancers.

Even though the photoelectric absorption process reaches its maximum probability when the excitation occurs at energies just above the absorption edge, in certain high-Z elements the maximum dose enhancement would not be close to these energies. This is explained by the need to find an energy that simultaneously maximizes the ratio of the mass energy absorption coefficients and the ratio of the mass absorption coefficients of the enhancing element with respect to the tissue to be doped [14,35]. In particular, in the case of gold, the maximum dose enhancement has been obtained at photon energies of 40 keV [36], which is considerably below its K-edge (80.7 keV). Nevertheless, in the case of iodine with a K-edge of 33.3 keV, the optimal energy has been determined both theoretically and empirically to be 50 keV [14]. In the particular case of gadolinium, unlike gold or iodine, the optimal energy described in the literature is 60 keV [14] or 65 keV [31], slightly above its absorption K-edge of 50.2 keV.

The determination of the Dose Enhancement Factor (DEF) has been addressed by different Monte Carlo (MC) simulations, as in the work of Zygmanski et al., 2013 [37], who performed a comprehensive study using the GEANT4 MC code with GNPs, testing different monoenergetic beams between 11 keV and 1 MeV and applying a water phantom with NPs located at a 2 cm depth. This work evaluated the dependence on the distance from the source to the GNP, beam size, NP size, and NP clustering, concluding that the DEF is sensitive to specific irradiation geometries and source types and essentially depends on the X-ray beam energy, which for energies under 20 keV resulted in beam attenuation rather than dose enhancement. Spiga et al., 2019 [38], using the same code, investigated the dose distribution of an X-ray beam slightly above the absorption edge of metal-based compounds (gadolinium and iodine) at several concentrations in a water-equivalent phantom. For monochromatic energies between 30 and 140 keV, comparing Monte Carlo data with the experimental results, strong agreement was obtained with differences of less than 5% for depths <60 mm. Regarding iodine dose enhancement, for 10 mg/mL concentrations and 35 keV spectra, increases of up to 150% in the dose were reported, reaching maximum enhancement for the 45 keV spectrum with a dose enhancement of 190%. In the case of gadolinium, at the same concentration, maximum enhancement was obtained for the 55–75 keV spectral range, with a difference of 40% at 21 mm and 20% at 54 mm compared to iodine. Another study reporting on Gd with monochromatic beams between 51 and 100 keV showed the existence of the DEF for energies above the K-edge, with a maximum DEF in the 58–65 keV range and a marked decrease for higher energies [29,31].

On the other hand, experimental quantification still presents important challenges, the main difficulty being the ability to record the contribution of low-energy photoelectrons and Auger electrons, which do not manage to pass through the walls that cover traditional dosimeters and be recorded by the radiosensitive region. Dose enhancement measurements obtained through Gafchromic model EBT2 and EBT3 radiochromic films (specially designed for “External Beam Therapy (EBT)” and commercially known as EBT2 and EBT3 films) have already been reported some years ago [39,40,41,42,43]. The EBT3 film possesses a 30 μm radiosensitive substrate placed between two 125 μm transparent polyester films, barriers not penetrable by electrons with energies <90 keV, making it difficult to directly measure the DEF for energy beams close to the Gd absorption edge. A feasible alternative is film delamination on one of the faces, exposing the polymeric substrate to the medium and the photon beam. The delamination process has been suggested and assessed for different beam types and qualities, such as alpha particles [44], medium-energy X-rays [45], and low-energy heavy-ion dosimeters [46], removing the energy constraint of the detectable particles. However, in these studies, EBT3 films were not submerged in water since the active substrate is highly sensitive and is easily damaged when in contact with water. This limits the dosimetric determination of dose enhancement at different concentrations since the experimental conditions are different from an actual clinical scenario. Consequently, interventions are limited to delamination followed by the application of a new waterproof element with a thickness that enhances the detection of low-energy electrons. Recently, the possibility of altering EBT3 dosimetric films by delaminating and resealing them using materials with thicknesses of 12 μm to maintain their water immersion properties has been reported [47]. The dosimeters evaluated after the intervention maintained their dosimetric properties unaltered, allowing the recording of photoelectrons of energies greater than 25 keV, generated by a ^192^Ir brachytherapy source in a phantom with Gd suspensions and dose enhancement values of 15%, compatible with Monte Carlo simulations and previous measurements.

The aim of this work was to find a method to optimize Gd-nanoparticle dose enhancement measurement, allowing one to record the dose contribution of photoelectrons with energies below 20 keV (typically produced in irradiation with electronic brachytherapy equipment). The use of delaminated and non-resealed EBT3 films and filters sufficiently thin to let through the generated photoelectrons, maintaining the property of being a barrier between the radiosensitive substrate and the water with the Gd suspension, is promising.

## 2. Materials and Methods

### 2.1. EBT3 Radiochromic Film Processing and Reading

Gafchromic^®^ EBT3 films (Ashland Advanced Materials, Bridgewater, NJ, USA) were meticulously delaminated based on the method described by [47]. The opening of the first 125 μm layer of polyester was carried out employing a surgical scalpel and applying a constant traction force achieved through the use of a controlled mechanical device.

From the delaminated EBT3 film sheets, 30 mm square fragments were obtained in order to be used as dosimetric elements adaptable to the implemented setup. All the delaminated EBT3 films used during the experiments were characterized in terms of their optical transmittance by pre- and post-irradiation readings, performed using an EPSON Perfection V370 (Seiko Epson Corporation, Suwa, Nagano, Japan) transmittance scanner, configured at 48 bits (16 bits per color) and 144 dpi. Additionally, since any interference with the film (including radiation exposure and mechanical stress) would alter the active substrate, producing polymerization reactions, a stabilization time of at least 16 h had to be allowed before any reading. Thus, the pre-irradiation reading was executed 24 h after the mechanical intervention (i.e., delamination and cutting), while a 24 h post-irradiation reading was chosen.

The data acquired with the scanner were processed with ImageJ software (Version 1.54), utilizing exclusively the red channel, since it has been widely reported that this channel provides the dose–response curve with the highest sensitivity for doses lower than 8 Gy. In view of the possible inherent measurement fluctuations that may have been registered in each pixel of the digital image, the same region of interest (ROI) size of 50 × 50 square pixels was defined for all the images analyzed. For this ROI, the “Measurement” function was used, which provided statistical data such as: maximum pixel value, minimum value, average, and standard deviation. In all measurements, the selected reading value was the average. To establish the change in the dosimeter transmittance due to the absorbed dose effect, the figure of merit used was the optical density (OD), as follows:

(1) $$D.O.=log\left(\frac{I_{0}}{I}\right);\;\Delta D.O.=\frac{1}{ln_{10}}\cdot \sqrt{\frac{\Delta {I_{0}}^{2}}{{I_{0}}^{2}}}+\frac{\Delta I^{2}}{I^{2}}$$

where $I_{0}$ is the average pixel intensity of an ROI of the pre-irradiation transmission image, and *I* is the post-irradiation reading averaged in the same ROI.

### 2.2. PMMA Phantom Manufacturing and Setup

A phantom that simulated a tumoral volume doped with a Gd suspension was designed, which allowed direct adaptation to the clinical electronic brachytherapy equipment applicator and the superficial irradiation of the volume. A second feature of the designed phantom was that one of its sides allowed the adherence of films of different thicknesses, without experiencing the leakage of the water with the Gd suspension. In this way, the phantom could be positioned directly on a delaminated EBT3 film, without the need to dip the film directly into the dose-enhancing medium.

**Figure 1:**
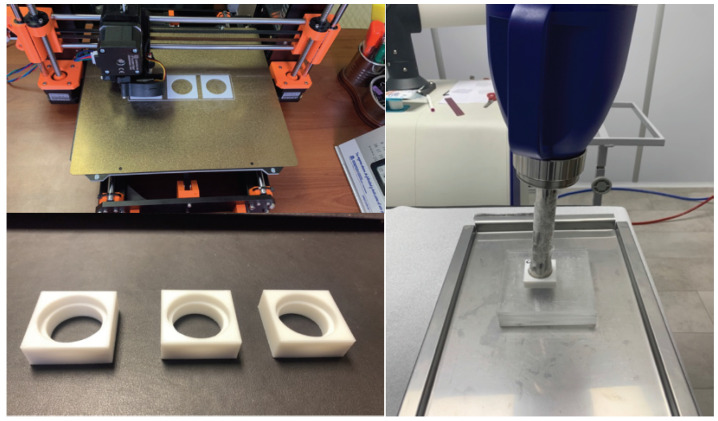
(**Left**) Design of PMMA phantoms by means of 3D printer. (**Right**) Applicator inserted into phantom.

The phantom was manufactured as a square prism with an external geometry of 4.5 cm for the square face side and 1.5 cm in height. Its interior was formed by two concentric cylinders, the first cylinder being 2.8 cm in diameter, which completely crossed the prism, enabling the phantom to be opened on both ends (Figure 1), and the second being 3.5 cm in diameter and 5.0 mm deep. The measurements were adapted to the D30-5 surface applicator diameter, permitting the applicator to rest on the phantom and keeping a constant distance of 1.0 cm from the irradiation surface to the other prism external face, where the EBT3 film would be placed behind the insulating film of the water with the Gd suspension (measured at a 1.0 cm depth in water) (Figure 1).

PMMA in the form of 3D printer filament, 1.75 mm in diameter, was selected as the fabrication material for the phantom walls. Thus, the phantom was directly printed using a PRUSA 3D printer model i3 MK3S (Figure 1). For the printing parameters, a 100% infill was selected in order to reduce the leakage of the water with the Gd suspension through the walls, maximizing the measurement time without damaging the delaminated EBT3 film on which the phantom was positioned. Multiple phantoms with the same geometrical characteristics but different extruder and bed temperature conditions were manufactured to evaluate the imperfections in the geometry and tolerances in the measurements considering the requirement of containing the clinical applicator and the Mylar film with the EBT3.

The fabricated phantoms were sealed on one side with a Spectro Film thin Mylar film marketed by SPI supplies (West Chester, PA, USA), presenting thicknesses of 2.5 and 12 microns. This allowed them to contain a total volume of 6.15 cm^3^, which was filled with Gd suspensions in concentration ranges of 0–30 mg/mL, simulating a tumor undoped and doped with Gd, and the Mylar films acted as two different energy barrier thicknesses for the photoelectrons produced by the Gd, which were recorded on the other side by the delaminated EBT3 film. Subsequent to the manufacture of the PMMA phantom, the entire structure was completed with a set of PMMA slabs (15 × 15 × 0.5 × 0.5) cm^3^ to achieve the backscattering component (see Figure 1).

Given that the Mylar film acted as a thin interface between the region with water containing the Gd suspension and the region with the radiosensitive substrate of the EBT3 dosimeters (which is highly affected in its dosimetric and structural properties by direct water contact), and given the inherent porosities that exist in 3D printing machining, it was necessary to evaluate the degree of phantom sealing. The implemented criteria considered maximum times without filtration under static conditions (time taken for the filter and the walls of the phantom to leak the suspension onto the EBT3 film while resting on it) and dynamic conditions (time taken for the filter and the phantom walls to leak the suspension onto the EBT3 film after the successive positioning and removal of the phantom on the film—conditions more similar to the required measurement process, which generated micro-tears in the Mylar film that would allow the leakage of water with the Gd suspension onto the EBT3 film in a shorter times).

### 2.3. Irradiation System

**Figure 2:**
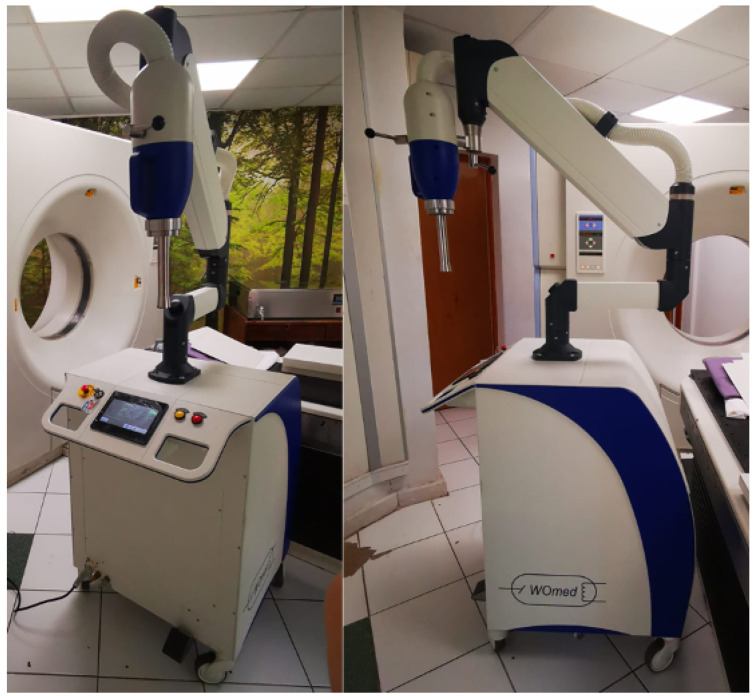
WOmed ioRT-50 clinical surface radiotherapy equipment used in the measurements.

Clinical treatment equipment consisting of a WoMed model IORT-50 mobile surface radiotherapy and intraoperative radiotherapy system was used (Figure 2). This equipment allows irradiation with 3 beam qualities, 35, 50, and 70 kVp, and maximum surface dose rates of 1–1.5 Gy/min. The system has a set of collimators that permit the formation of circular fields 1–3 cm in diameter on the surface for surface irradiation and a set of spherical applicators 3–5 cm in diameter for intra-operative treatments. Depending on the type of applicator, the system configures a kVp and a current for which it has dosimetric calibration tables at different water depths. In this way, the irradiation time is selected exclusively according to the dose to be prescribed in the treatment. Given the need to minimize radiation penetration for surface treatments, the equipment in its treatment mode allows the use of energies of 35 and 50 kVp exclusively, limiting the beam quality to 70 kVp for intra-operative treatment only.

When employing EBT3 films as dosimeters, it is imperative to generate a flat irradiation on them in order to use the information from the largest number of pixels. This creates the need to exclusively implement the surface applicator (which is why it was necessary to design the phantom to be compatible with this accessory, as described in the previous subsection) and operate the equipment in “Service” mode, for the purpose of removing the kVp restriction, allowing a 70 kVp beam quality to be used with this applicator. However, since the equipment does not have depth dose tables for this beam quality, it was necessary to first perform dosimetric measurements to determine the dose rate delivered at that energy with the applicator. Dosimetric determination was performed following the TG-61 code of practice for the 50 kVp (HVL: 3.38 mm Al) and 70 kVp (HVL: 4.70 mm Al) beam qualities [48], whose kV were verified according to routine quality-control equipment using a RadCal Accu-kV (40–160 kVp) kV meter with a valid calibration certificate. The dosimetric protocol involves obtaining the dose from in-air kerma measurements taken within a small-volume parallel-plate ionization chamber calibrated under these conditions. A PTW model TN34013 chamber with a sensitive volume of 0.005 cm^3^, designed for measurements in the 10–70 kVp range, and a PTW model Unidos E electrometer were used for the measurements.

### 2.4. EBT3 Film Calibration Curve

The determination of dose enhancement by the dosimetric use of EBT3 films requires the prior acquisition of a calibration curve that relates the change in optical density to different absorbed doses in the ranges of interest and under the same conditions as the experimental procedure: at the prescription depth (1 cm) and placing the radiochromic film under a PMMA phantom with water. Reproducing the same reference conditions for calibration and measurement is fundamental for the range of kV energies, since unlike MV beams, the former drastically change their quality at different depths in a medium. The calibration curve was obtained considering 6 measurement points in the dose range of 1.5–5.5 Gy, with each measurement in triplicate (18 total measurements), in order to consider both the uncertainties of the procedure and the heterogeneity inherent to the EBT3 film. The resulting curve was obtained by fitting a polynomial function, using the R^2^ parameter as a goodness-of-fit criterion.

### 2.5. Dose Enhancement Evaluation

Dose enhancement was measured in an initial stage considering two available beam qualities: one with an energy below the K absorption edge of Gd (50.2 kV) of 50 kVp, for which it was feasible to generate dose enhancement in the L and M layers of the Gd exclusively; and one with an energy of 70 kVp, above the K absorption edge, which could additionally generate dose enhancement in the K layer. Two concentrations of water with a Gd suspension at 10 and 20 mg/mL were prepared and injected into the PMMA-fabricated tumor phantom. A set of 10 measurements was performed for each concentration and for each beam quality to account for possible fluctuations in the film. The prescription in each measurement was 3.0 Gy in water at the depth of the EBT3 film (1.0 cm), which was achieved by adjusting only the irradiation time, since for both configurations (50 kVp and 70 kVp) a fixed current of 7 mA was used. As a barrier between the Gd suspension and the active substrate of the delaminated EBT3 film, a 2.5 micron Mylar filter was placed on the tumor phantom, which limited the penetration of photoelectrons with an energy less than 10 keV. The dose enhancement was evaluated from the DEF defined as a function of the beam quality E (in this case, kVp) and the percentage of Gd (concentration in mg/mL), presenting uncertainty values of one SD for all measurements:

(2) $$DEF_{\left(E,\%Gd\right)}=\frac{\mathrm{Dose}\;\mathrm{readout}\;\mathrm{in}\;\mathrm{EBT}3\;\mathrm{for}\;\mathrm{beam}\;\mathrm{quality}\;E\;\mathrm{and}\;\mathrm{phantom}\;\mathrm{with}\;\mathrm{Gd}}{\mathrm{Dose}\;\mathrm{readout}\;\mathrm{in}\;\mathrm{EBT}3\;\mathrm{for}\;\mathrm{beam}\;\mathrm{quality}\;E\;\mathrm{and}\;\mathrm{phantom}\;\mathrm{without}\;\mathrm{Gd}}$$

Considering that beam qualities slightly above the absorption edge (such as that of 70 kVp emitted by the studied clinical equipment) allow one to achieve a dose enhancement at greater depths than lower energies, it was decided to study the concentration at which the competitive phenomenon of dose decrease due to self-attenuation (the attenuation of the incident radiation by the high Gd concentration) would offset the increase in dose due to the enhancement phenomenon, in order to determine the range of useful concentrations. Additionally, given that there will always be a sub-quantification of the net dose enhancement, given the inherent restriction of photoelectrons with a lower energy imposed by the Mylar coating necessary to isolate the delaminated EBT3 film from direct contact with the water containing the Gd suspension, we proceeded to evaluate the dose achieved by the different concentrations for two Mylar film thicknesses: 2.5 μm and 12 μm. The latter thickness corresponds to a barrier that restricts the detection of all the photoelectrons produced by the K-edge, since it imposes an energy barrier of 25 keV.

Three different concentrations of water with a Gd suspension were prepared: 10, 20, and 30 mg/mL. A volume of 6.15 cm^3^ of each concentration was infused inside 3 PMMA phantoms, closed at one end with a 2.5 μm Mylar film (minimum thickness available as a barrier for the generated photoelectrons). Similarly, a volume of 6.15 cm^3^ of each concentration was infused into 3 PMMA phantoms that were closed at one end by a 12 μm Mylar film (a thickness that would not allow the penetration of photoelectrons with energies less than 25 keV). A prescription of 3.0 Gy in water was delivered at a depth of 1.0 cm (the position of the delaminated EBT3 film), with the equipment configured at 70 kV and 7 mA and an irradiation time of 1 min and 6 s. Differences in the doses recorded for the same beam quality and for the same Gd concentration, but with a phantom equipped with different Mylar thicknesses, were associated exclusively with the dose enhancement component, which was recorded as significantly higher in the measurements of the 2.5 μm Mylar film than those of the 12 μm Mylar film, allowing at first order the separation of the dose enhancement from the attenuation components of the total dose measured.

## 3. Results

### 3.1. PMMA Phantom Manufacturing and Evaluation

**Figure 3:**
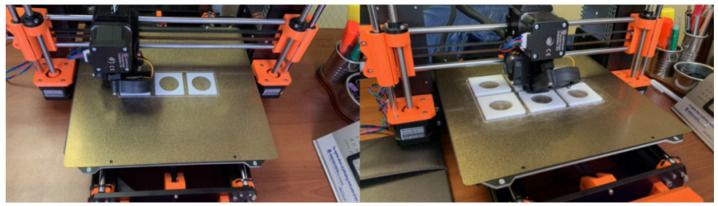
Manufacturing of multiple phantoms with the same geometry but different temperature parameters for the nozzle extruder and printing surface.

Following the defined geometries and the tolerances given by the clinical equipment used, it was possible to manufacture by the additive deposition of PMMA filaments multiple versions of the phantom adaptable to the surface applicator to contain the Gd suspension (Figure 3). Given the particular characteristics of the PMMA material in filament form, it was necessary to evaluate the results of the final prints of the phantom in terms of geometry imperfections and tolerances in the final measurements obtained for different temperature values of the printer extruder, in the range of 230–250 °C, indicating that the optimum temperature was 250 °C. Additionally, the same criteria were compared for different printed phantoms depending on the temperature of the printing surface, in the range of 90–110 °C, indicating that the best results were achieved for 100 °C.

**Figure 4:**
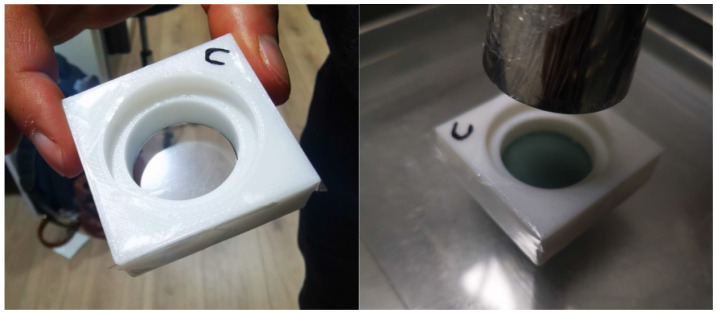
PMMA phantoms sealed with 2.5 μm and 12 μm Mylar films to contain a 6.15 cm^3^ suspension volume deposited on delaminated EBT3 film dosimeters.

From the passive and active tightness criteria defined for the PMMA phantom sealed with 2.5 μm or 12 μm Mylar filters (Figure 4), it was determined that for the case of phantoms configured with a 12 μm Mylar film, the passive tightness reached average times of up to 30 min without filtering, with the first affected area being the edges of the EBT3 film. Likewise, phantoms configured with a 2.5 μm Mylar film resisted for average times of 25–30 min. A relevant aspect to consider is that, in real measurement conditions, the phantoms are repeatedly positioned on different films for shorter irradiation times, with the actions of positioning and removing inducing more damage on the thin film, resulting in the generation of micro-cracks capable of causing the leakage of the water with the Gd suspension. When implementing the active sealing evaluations, it was evidenced that the average sealing time was effectively reduced by half, and the first leaks for both Mylar thicknesses occurred after only 15 min. However, given the equipment dose rate, the irradiation times required for the calibration curves and dose enhancement measurements were lower than the active sealing times, ensuring that the phantom configuration used was adequate for the required measurement.

### 3.2. Dose Enhancement Measurements

#### 3.2.1. Calibration Curve

**Figure 5:**
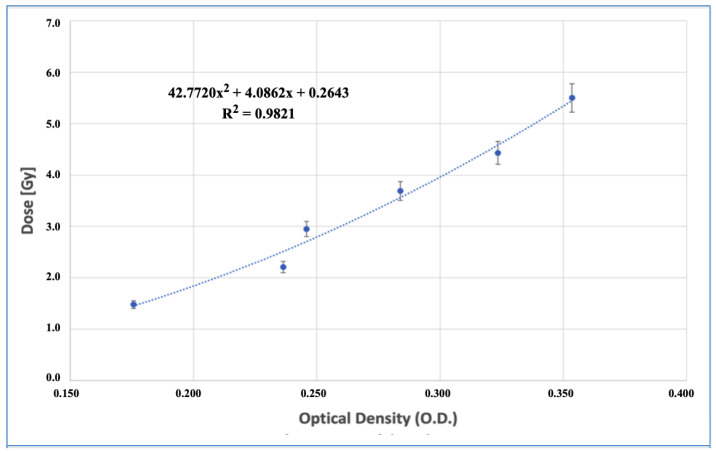
Delaminated EBT3 film dose–response curve in terms of optical density achieved at different dose values in the range of 1.5–5.5 Gy.

Figure 5 displays the calibration curve obtained from fitting the dose-optical density response of the delaminated films read 24 h post-irradiation to a second-degree polynomial function. The fit quality achieved for the dose range of 1.5–5.5 Gy was R^2^ = 0.9821. The error bars correspond to the dispersion value obtained in triplicate for each point on the curve. The indicated uncertainties correspond to one SD level.

Is important to note that the OD–dose relationship is not linear in EBT3 films, and better results are achieved through empirical models with variable coefficients or polynomial fits. Besides, the OD values are only valid for a specific wavelength of light analyzed [49] and can vary according to the scanner and parameters like the scanner orientation and reading conditions [50].

#### 3.2.2. Dose Enhancement Evaluation as a Function of Beam Quality

For the first evaluation of the DEF, in terms of beam quality and a dose prescription of 3.0 Gy at a 1.0 cm depth, it was found that for irradiations with 50 kVp at concentrations of 10 mg/mL Gd, there was a decrease in the prescribed dose, while the same behavior was exhibited for the higher concentration of 20 mg/mL, as shown in Table 1. This phenomenon could be explained given that the 50 kVp beam was not able to excite the K-edge of Gd (50.2 keV edge), so it only had the option of generating enhancement in the L layer. However, the greater beam attenuation achieved when propagating in a medium with a higher attenuation coefficient than water generated a more substantial effect compared to dose enhancement, which was accentuated at higher concentrations, not allowing dose enhancement to be achieved at the concentration and depth studied.

For the 70 kVp beam quality, a slightly higher dose was obtained for irradiations at 10 mg/mL Gd concentrations. On the other hand, for the 20 mg/mL Gd concentration, an increase of a 22±7% DEF was achieved (Table 1). For this beam quality, it was observed that the contribution of photoelectrons generated in the photoelectric absorption processes with the K layer was more significant than the contribution to the dose from interactions with the L layer, although only photoelectrons with energies greater than 10 keV were recorded with the 2.5 μm film. For this reason, only the limited spectral region between 60 and 70 keV of the radiation beam contributed to the dose enhancement in the dosimeter. Additionally, the higher energy decreased the self-attenuation generated by the higher-absorption-coefficient medium, allowing, at least at the tumor depth studied, the enhancement phenomenon to be greater than the attenuation, achieving an effective DEF.

#### 3.2.3. Dose Enhancement Evaluation as a Function of Gd Concentration for the Most Energetic Clinical Beam Quality

The results obtained from the readings of the new film set (considering a 70 kVp energy and a Gd concentration range between 10 and 30 mg/mL), which were transformed to dose via the calibration curve previously obtained, once again showed dose values equivalent to those previously obtained for 10 and 20 mg/mL Gd concentrations of 3.17±0.17 Gy and 3.67±0.22 Gy, respectively. For the newly evaluated concentration of 30 mg/mL, an average DEF of 9±7% was recorded (Table 2), which was slightly above the experimental uncertainty, possibly marking the concentration limit at which dose enhancement would generate a net dose increase at the studied depth, and thus the optimal concentration value to use would be around 20 mg/mL.

As mentioned, there is a point in a dosimeter where the dose enhancement does not predominate and the dose begins to fall due to the attenuation produced by the high concentrations of the enhancement material. With the purpose of evaluating, at first order, the percentage of attenuation and the percentage of dose enhancement contribution separately with respect to the net dose recorded, the delaminated EBT3 films with a 12 μm Mylar barrier (which completely eliminated the enhancement component of the K-edge) were assessed. A dose of 2.84±0.15 Gy was obtained for the 20 mg/mL concentration of Gd, equivalent to a decrease of 6±5% in the prescribed dose (a consequence of the higher attenuation with respect to water), while for the 30 mg/mL concentration a dose of 2.43±0.18 Gy was obtained, corresponding to a decrease of 19±6% in the prescribed dose (Table 3).

The results obtained showed more significantly that although the concentration increase generated a strong attenuation, the dose enhancement component of the K-layer was responsible for generating a significant additional dose proportional to the concentration, which in the case of the 20 mg/mL Gd concentration produced an additional dose increase of 29±7% and for the 30 mg/mL concentration an increase of 34±7% (Table 3).

## 4. Discussion

Previously reported methods found in the literature that involve the delamination and subsequent resealing of EBT3 films limited the dosimetry measurement in water of the dose enhancement produced by nanoparticles to exclusively gamma photons and medium-energy X-rays (>80 kV according to the IAEA TRS-398 protocol), which were capable of generating photoelectrons of energies greater than 25 keV that managed to pass through the thin film used in the resealing process (10-times thinner than the original laminate) [47], preventing the use of this methodology for the low-energy beams (<80 kV) employed by eBT and IORT units. The method proposed in this work used a filter that was five-times thinner (2.5 μm) than that of the previous procedure, since its function was limited to being a waterproof barrier between the radiosensitive substrate present in the film and the water that contained the Gd suspension, thus removing the mechanical strength and elasticity that was required of the filters, in both the resealing process and the subsequent immersion of the film in water, damaging the latter due to the entry of water into the active substrate and resulting in a greater dispersion and uncertainty in its reading. In this way, by reducing the thickness of the film, the photoelectron detection range was increased to energies of 10 keV and higher, and the mechanical stress effects that occur due to resealing and submerging the film in water were removed.

The assays performed using PMMA phantoms manufactured by 3D printing with infill percentages of 99% and a 2.5 μm Mylar film accession showed an adequate ability to hold water with a Gd suspension at a volume of 6.15 cm^3^ without leakages for 15 min, considering rise and fall movements. In addition, it should be noted that the existence of 3D-printed filaments of dosimetrically useful materials such as PMMA permits the fast, customized, and economical generation of phantoms adaptable to unconventional requirements such as dose enhancement measurements.

For the clinical equipment and beam quality used (70 kVp, HVL 4.70 mm Al), the greatest dose enhancement for the gadolinium suspension was obtained for a 20 mg/mL concentration, with an increase of up to 22±7% with respect to the prescribed dose, while for 30 mg/mL concentrations only a 9±7% enhancement was achieved, due to the existence of a critical competitive process between concentration and dose enhancement. For lower concentrations, it was not possible to discriminate the enhancement from the experimental uncertainties.

The evaluation of how the dose increase was modified as a function of the Mylar film thickness imposed onto the EBT3 dosimeter showed that by implementing a thickness that blocked the totality of the photoelectrons generated from the Gd K-edge, the doses for the 20 mg/mL concentration decreased by 6±5%, while for 30 mg/mL concentrations the decrease in the dose reached 19±6%. This shows that the photoelectrons contributed by the K-layer that managed to pass through a 2.5 μm thick Mylar film promoted dose increases of 28±7% and 34±7% for 20 and 30 mg/mL Gd concentrations, respectively. Thus, it is expected that implementing Mylar films of smaller thicknesses or dosimeters without these limitations would result in much higher dose enhancement measurements than those reported in this work.

Even though the obtained results were a sub-estimation of the actual dose enhancement, since we were not able to detect photoelectrons of an energy lower than 10 keV, increasing the energetic recording range has shown a significant advancement in the feasibility of measuring dose enhancement at first order produced by the K-edge of Gd in a series of existing electronic brachytherapy and intraoperative radiotherapy clinical units, approaching values reported by Monte Carlo simulations.

## 5. Conclusions

It is feasible to implement delaminated EBT3 films without resealing by manufacturing adequate phantoms that can contain the necessary volume of water with a Gd suspension and adding films of reduced thickness that act as walls and prevent the leakage of the content.

The set of printed PMMA phantoms and the access to Mylar filters of smaller thicknesses open up the possibility of implementing these measurements in different clinical systems, in order to evaluate optimal configurations for various beam qualities and high-Z agents.

We performed an experimental determination of the Dose Enhancement Factor for a 70 kVp beam quality produced by a clinical electronic brachytherapy system, showing a maximum dose enhancement for a gadolinium suspension concentration of around 20 mg/mL, while a decrease was observed for higher concentrations due to the self-shielding effect that the same Gd nanoparticles generated in the incident radiation spectra.

## Figures and Tables

**Table 1:** Dose and DEF achieved for 50 and 70 kVp beam quality with 10 and 20 mg/mL Gd concentrations.

Medium	50 kVp		70 kVp	
	Dose (Gy)	DEF	Dose (Gy)	DEF
Water	3.00	–	3.00	–
10 mg/mL	2.84 ± 0.12	−5 ± 4%	3.16 ± 0.18	+6 ± 6%
20 mg/mL	2.32 ± 0.18	−23 ± 6%	3.67 ± 0.21	+22 ± 7%

**Table 2:** Dose and DEF achieved for 70 kVp beam quality with 10, 20, and 30 mg/mL Gd concentrations.

Concentration (mg/mL Gd)	Prescribed Dose (Gy)	Measured Dose (Gy)	DEF (%)
10	3.00	3.17 ± 0.17	+6.0 ± 6%
20	3.00	3.67 ± 0.22	+22.0 ± 7%
30	3.00	3.26 ± 0.20	+9.0 ± 7%

**Table 3:** Dose recorded for Mylar films that prevented enhancement recording (12 μm) and Mylar films that allowed enhancement recording (2.5 μm) for a beam quality of 70 kVp at Gd concentrations of 20–30 mg/mL.

Concentration (mg/mL Gd)	Prescribed Dose (Gy)	Measured Dose with a 12 μm Film (Gy)	Measured Dose with a 2.5 μm Film (Gy)	Relative DEF (%) 2.5 μm/12 μm
20	3.00	2.84 ± 0.15	3.67 ± 0.22	+29.0 ± 7%
30	3.00	2.43 ± 0.18	3.26 ± 0.20	+34.0 ± 7%

## Data Availability

Data are contained within the article.
